# Electronic Structure and Magnetic Properties of a High‐Spin Mn^III^ Complex: [Mn(mesacac)_3_] (mesacac=1,3‐Bis(2,4,6‐trimethylphenyl)‐propane‐1,3‐dionato)

**DOI:** 10.1002/cphc.202200652

**Published:** 2022-12-14

**Authors:** Nina M. Strassner, Sergej Stipurin, Primož Koželj, Yuri Grin, Thomas Strassner

**Affiliations:** ^1^ Faculty of Chemistry and Food Chemistry Physical Organic Chemistry Technical University Dresden 01062 Dresden Germany; ^2^ Department of Chemistry and Pharmacy Friedrich-Alexander-University Erlangen-Nuremberg 91054 Erlangen Germany; ^3^ Max-Planck-Institute for Chemical Physics of Solids 01187 Dresden Germany; ^4^ Jožef Stefan Institute 1000 Ljubljana Slovenia

**Keywords:** manganese (III) high-spin, magnetism, Jahn–Teller distorsion, density functional calculations, X-ray structure

## Abstract

Metal acetylacetonates of the general formula [M(acac)_3_] (M^III^=Cr, Mn, Fe, Co) are among the best investigated coordination compounds. Many of these first‐row transition metal complexes are known to have unique electronic properties. Independently, photophysical research with different β‐diketonate ligands pointed towards the possibility of a special effect of the 2,4,6‐trimethylphenyl substituted acetylacetonate (mesacac) on the electron distribution between ligand and metal (MLCT). We therefore synthesized and fully characterized the previously unknown octahedral title complex. Its solid‐state structure shows a Jahn‐Teller elongation with two Mn−O bonds of 2.12/2.15 Å and four Mn−O bonds of 1.93 Å. Thermogravimetric data show a thermal stability up to 270 °C. High‐resolution mass spectroscopy helped to identify the decomposition pathways. The electronic state and spin configuration of manganese were characterized with a focus on its magnetic properties by measurement of the magnetic susceptibility and triple‐zeta density functional theory (DFT) calculations. The high‐spin state of manganese was confirmed by the determination of an effective magnetic moment of 4.85 μ_B_ for the manganese center.

## Graphical abstract

**Synthesis and characterization of an octahedral manganese(III) complex** with sterically demanding 1,3‐bis(2,4,6‐trimethylphenyl)propane‐1,3‐dionato (mesacac) ligands is reported. Density functional calculations and experimental results agree on t^3^_2g_e^1^_g_ high‐spin state (S=2) showing a strong Jahn–Teller elongation. The electronic structure is also confirmed by SQUID‐magnetometer measurements where a magnetic moment of 4.85 μ_B_ is associated with the metal centre.

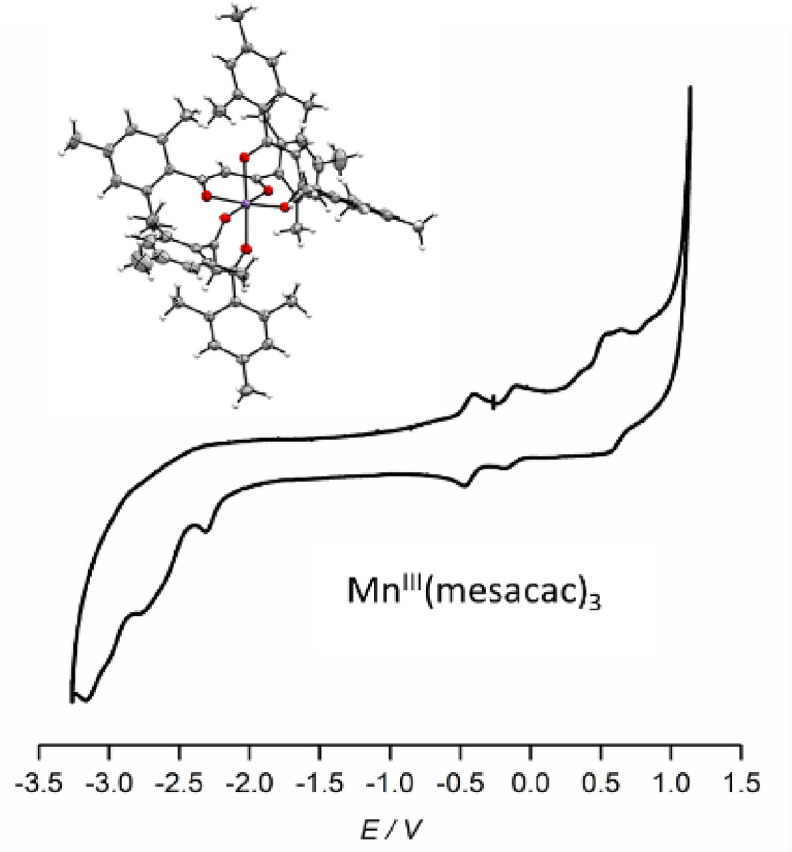

## Introduction

Pentane‐2,4‐dione or acetylacetone is the most well‐known member of a family of compounds which are often referred to as β‐diketones. These are the precursors of ligands (L) which literally can be found in complexes with all elements of the whole Periodic Table: the bidentate, negatively charged, acetyl‐acetonato (acac) ligands.^[1]^ Interest in beta‐diketonate metal complexes dates back to the 19th century,^[2]^ and especially the transition metal (TM) complexes have been of interest for both basic and applied research.^[3]^ TM(acac)_3_ compounds are usually characterized by an octahedral arrangement of the six oxygen atoms around the TM, and their structures have been investigated by different methods. Among these complexes, the high‐spin (HS) Mn(acac)_3_ has been of special interest due to its properties as a catalyst^[4]^ and radical initiator for polymerization reactions,^[5]^ and its ability to easily change oxidation states. It has been synthesized by different routes, from manganese(III) salts, but also directly from manganese(VII), as well as by comproportionation from Mn(II) and Mn(VII), which demonstrates the availability of a wide variety of oxidation states.^[6]^

The Mn(acac)_3_ structure^[7]^ shows a strong Jahn‐Teller distortion,[^7f^, ^8^] which has been the subject to many theoretical analyses by different methods.^[9]^ Due to the more recent interest in spin–crossover compounds^[10]^ also the spin states[^9c^, ^11^] of manganese(III) complexes have been investigated. Although most complexes show an t^3^_2g_ e^1^_g_ electronic configuration, there are examples with intermediate‐spin (IS)^[12]^ and also with low‐spin (LS) state^[13]^ like the hydrotris(pyrazolyl)borate manganese(III) complexes.^[14]^ This caught our attention as these ligands obviously have a strong effect on the order of the different orbitals and states, a quality we also need in our quest to improve the photophysical properties of organometallic complexes with the goal of finding the ultimate blue phosphorescent OLED emitter. We have recently reported phosphorescent platinum complexes with bis(pyrazolyl)borate ligands^[15]^ which showed very good properties and had previously worked on cyclometalated platinum and iridium β‐diketonate complexes.[^15a^, ^16^] In the latter the sterically very demanding 1,3‐bis(2,4,6‐trimethylphenyl)propane‐1,3‐dionato (mesacac) ligand also had an extremely beneficial effect on the electronic structure of the resulting metal complexes. According to the literature on manganese(III) β‐diketonate complexes, for the most part only the readily available beta‐diketonate ligands with alkyl or hexafluoro‐alkyl substituents have been used.^[9a]^ In some cases also the 1,3‐diphenyl‐1,3‐propanedionato (dibenzoylmethane, dbm) as well as 4‐halo or 4‐alkoxy substituted aromatic ligands,^[17]^ but we were surprised to see that the Mn^III^(mesacac)_3_ complex was not known. We therefore set out to synthesize this compound to investigate the effect of the ligand on the electronic structure of the manganese complex.

## Results and Discussion

### Synthesis of [Mn(mesacac)_3_]

**Scheme 1:**
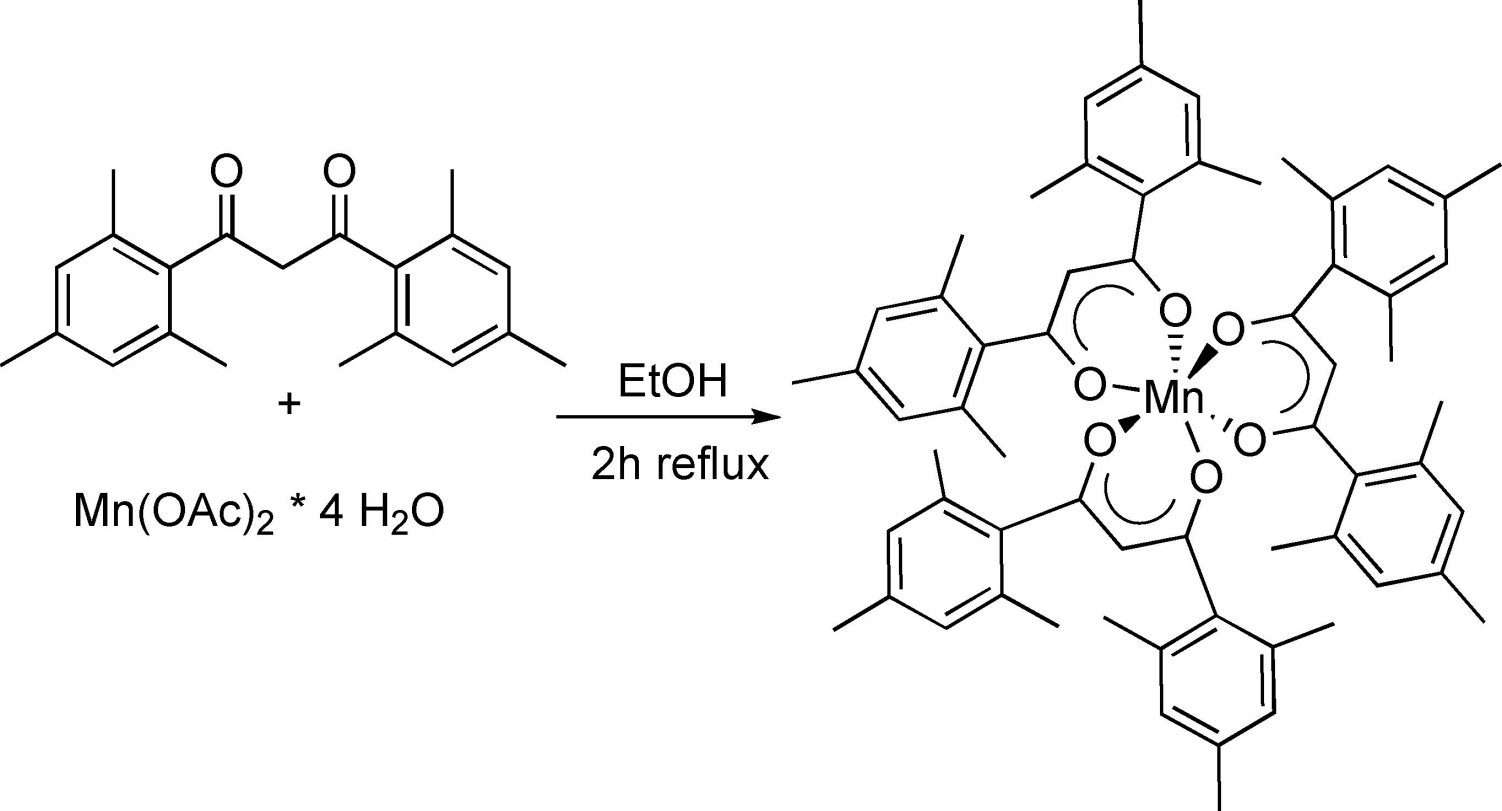
Synthesis of the [Mn(mesacac)_3_] complex.

The tris(1,3‐bis(2,4,6‐trimethylphenyl)propane‐1,3‐dionato‐κO^1^, κO^3^)manganese(III) [Mn(mesacac)_3_] complex was prepared by adapting a published procedure^[18]^ from the manganese(II) acetate tetrahydrate and the mesacacH ligand in 150 ml ethanol in air (Scheme 1).

### Electronic Absorption Spectroscopy

**Figure 1:**
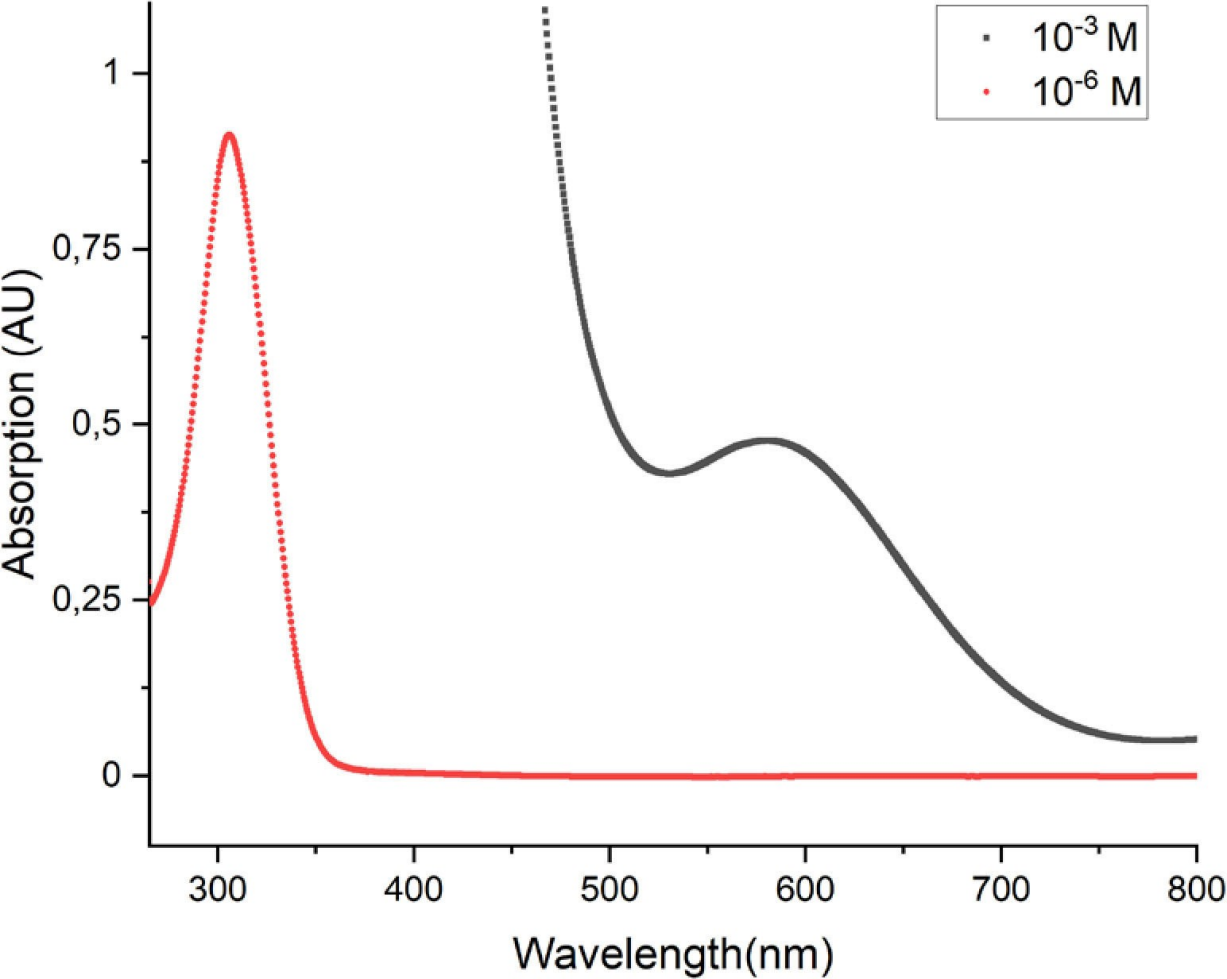
Quantitative absorption spectra of [Mn(mesacac)_3_] recorded in DMF at room temperature.

The photophysical characterization of the complex included absorption spectroscopy at room temperature (Figure 1). The absorption properties were recorded in dimethylformamide (DMF) solutions for two different analyte concentrations (10^−6^ M, Figure S1 and 10^−3^ M, Figure S2).

Both spectra show a strong absorption band at around 310 nm, with an intensity far above the generally desired values. The highly diluted solution (10^−6^ mol L^−1^) however gave access to a peak with a molar extinction coefficient of ϵ=9.1×10^6^ at 306 nm (according to the Lambert‐Beer law). This very strong absorption is caused by a d→π metal‐to‐ligand charge‐transfer (MLCT), similar as in the manganese (III) acetylacetonate Mn(acac)_3_ complex.^[19]^ The spectrum of [Mn(mesacac)_3_] recorded at high analyte concentration of 10^−3^ mol L^−1^ showed an additional absorption band at 600 nm. The low intensity (ϵ=4.8×10^3^) d→d transition in the octahedral complex is most likely caused by the Jahn‐Teller distortion.

### High‐Resolution Mass Spectrometry

**Figure 2:**
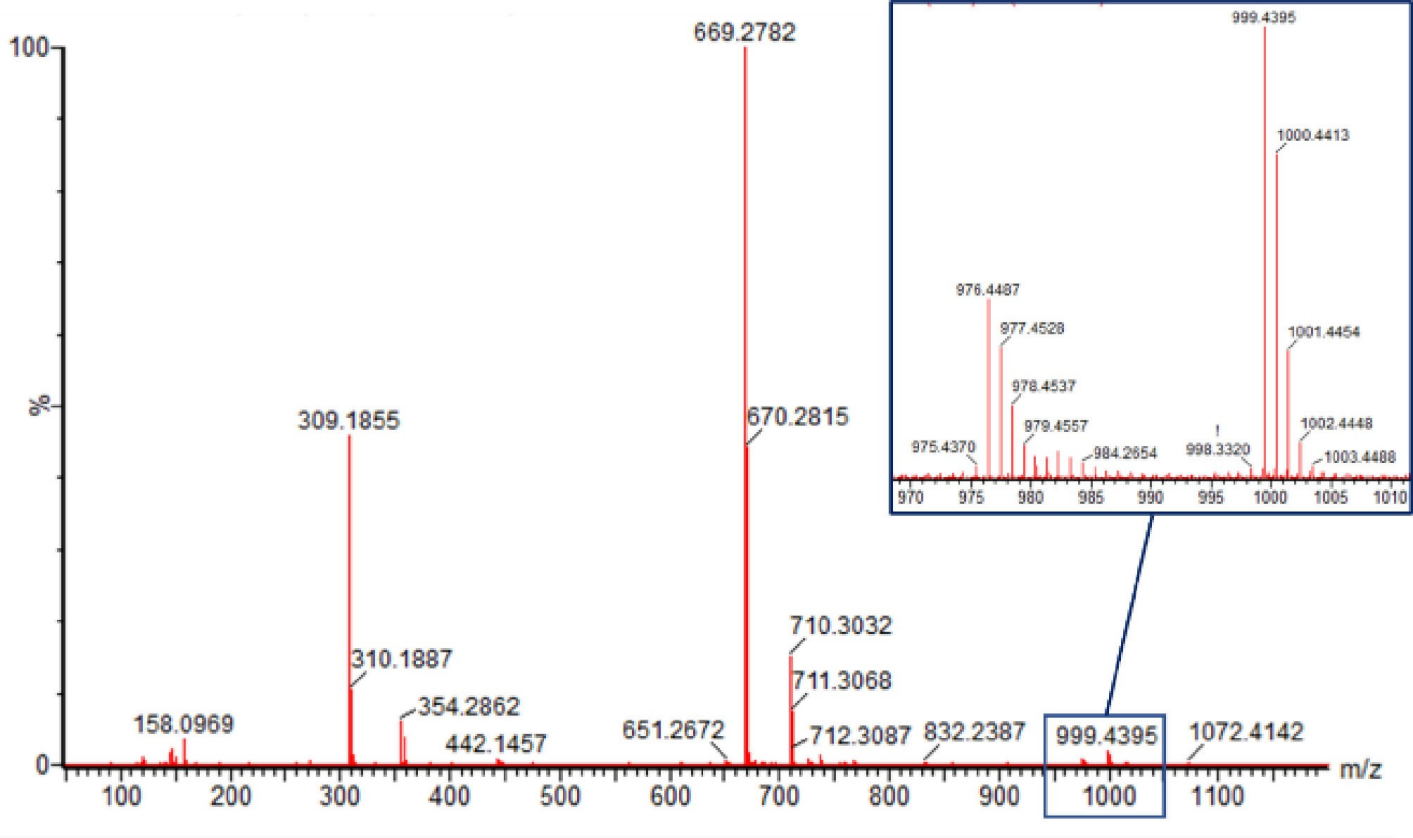
High‐resolution mass spectrum of Mn(mesacac)_3_.

The mass spectrum of Mn(acac)_3_ has been studied by regular mass spectrometry.[^19^, ^20^] We were interested in the high‐resolution mass spectrum of the title compound (Figure 2), especially as manganese only has one relevant stable isotope. Frequently, molecular cations [M^+^] cannot be detected in mass spectrometry due to immediate fragmentation of the ionized compound. The title compound, however, not only shows the molecule cations of the type [M+H^+^] at 977.4528 m/z typical for the ESI ionization technique, but also the [M^+^] ion at 976.4487 m/z resulting from electron loss. As seen in Figure 2, two very strong signals of fragments can be observed, which are both related to the loss of one mesacac ligand from the title complex. The fragment with a molecular weight of 669.2782 m/z is the remaining Mn(mesacac)_2_^2+^ complex, while the signal at 309.1855 m/z corresponds to the protonated ligand H_2_mesacac^+^ (Figure 2).

### Thermogravimetric Analysis

**Figure 3:**
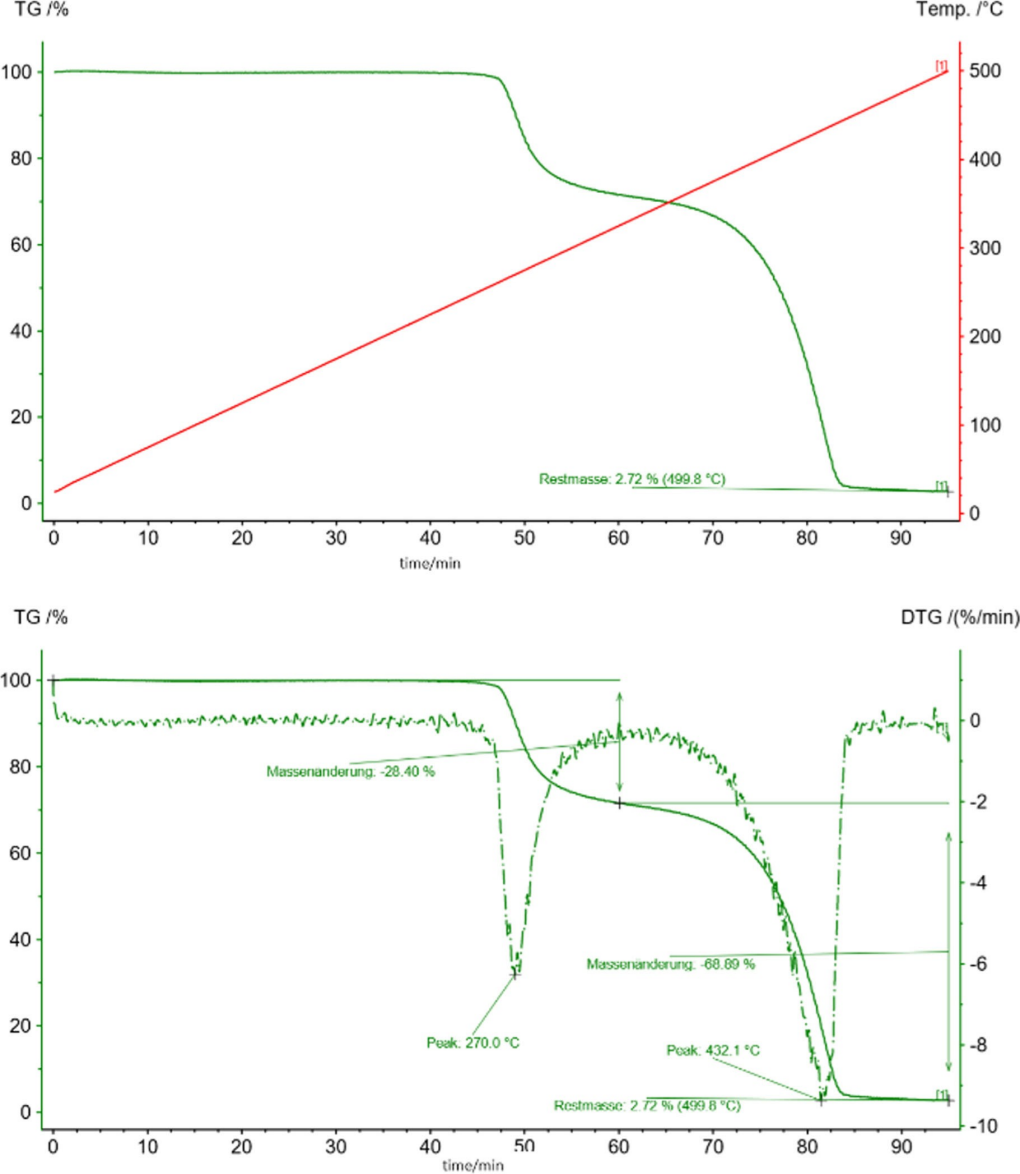
Thermogravimetric behaviour of Mn(mesacac)_3_, measured under argon, shows full stability until 270 °C. We independently heated two samples to 300 °C and 500 °C and then submitted these samples to magnetic measurements. The TGA, where we heated the sample up to 300°C only showed the first decomposition peak.

Additionally, the thermal stability of the complex was investigated by thermogravimetric analyses in an argon atmosphere (TGA, Figure 3). Compared to the manganese acetylacetonate complex Mn(acac)_3_ with methyl groups (instead of the mesityl groups), which has been reported to decompose as early as 150 °C,^[21]^ this new complex is thermally highly robust. No decomposition was observed below 270 °C, where a first mass loss of 28 % was observed. This can be attributed to the loss of a Mes‐propenyl‐Mes fragment from breaking the carbon‐oxygen bonds.

From additional magnetic measurements it became clear that the resulting complex is a manganese (II) complex, which is stable until full decomposition at 432 °C.

### Crystal Structure Determination

**Figure 4:**
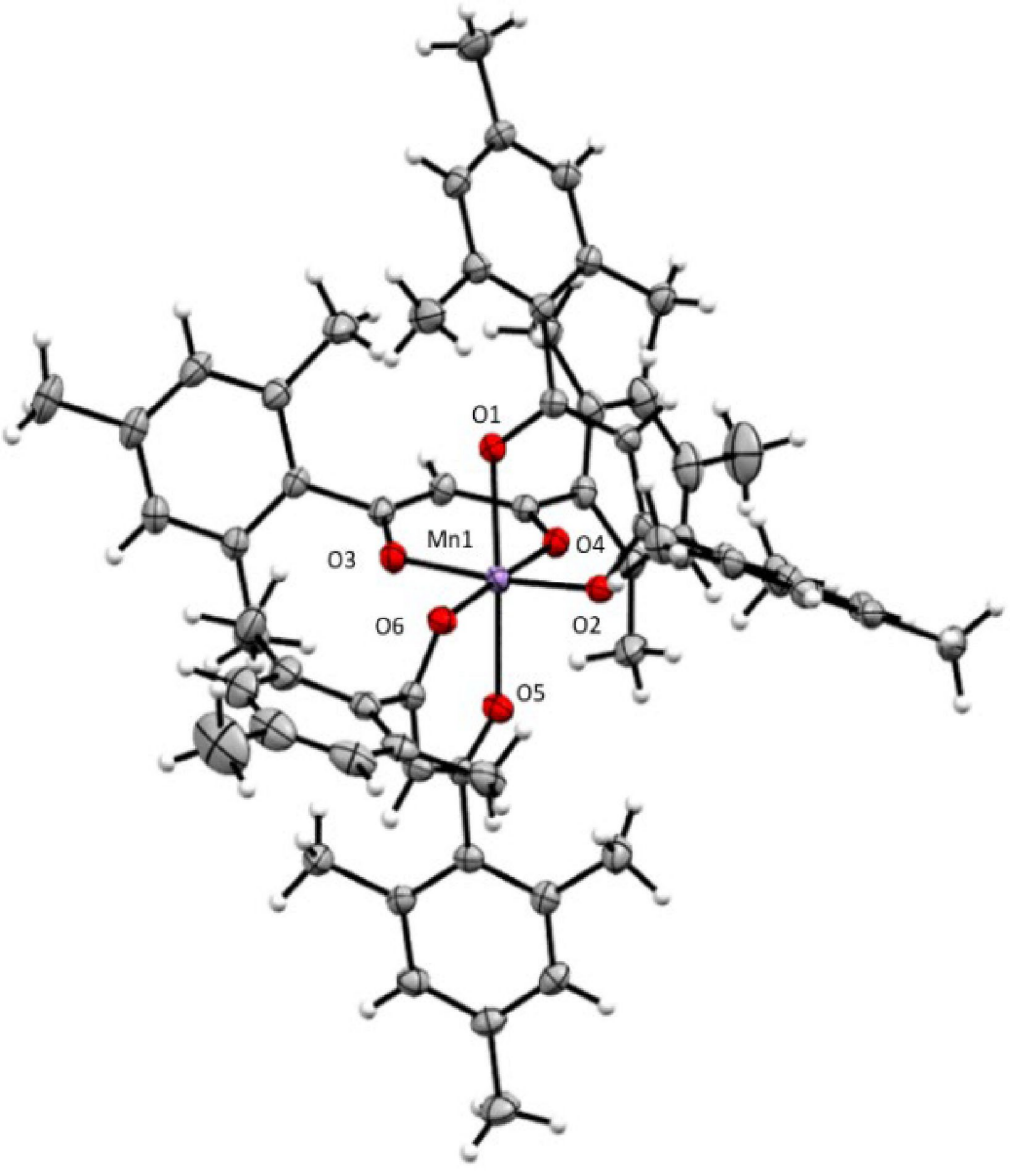
Crystal structure of Mn(mesacac)_3_. Thermal ellipsoids are drawn at the 50 % probability level. Selected bond lengths (Å) and angles (°): Mn1−O1 2.116(1), Mn1−O2 1.9270(9), Mn1−O3 1.934(1), Mn1−O4 1.9209(9), Mn1−O5 2.145(1), Mn1−O6 1.9177(9), O1−Mn1−O2 88.52(4), O1−Mn1−O4 87.57(4), O2−Mn1−O4 89.43(4); O2−Mn1−O6 89.63(4).

The crystal structure of the title compound (Figure 4) was obtained from a X‐ray diffraction measurement at a temperature of 110 K. The crystallographic information is provided in Tables S1 and S2 in the Supporting Information. Similar to the manganese (III) acetylacetonate complex Mn(acac)_3_, also the larger Mn(mesacac)_3_ crystallizes in the monoclinic space group of *P*2_1_/*n* with the unit cell containing four molecules.^[22]^ The manganese center is surrounded by six oxygen atoms with two Mn−O distances of 2.116(1) Å and 2.145(1) Å along the z‐axis and four equatorial bond lengths between 1.9177(9) Å and 1.934(1) Å. Complexes with d^4^ electronic configuration are well known for their Jahn‐Teller distortion, which was observed for Mn(acac)_3_[^7a^, ^7b^, ^8b^] before, and is also clearly visible in the case of the Mn(mesacac)_3_. The octahedral elongation compared to the different Mn(acac)_3_ structures known in the literature is slightly more pronounced. Nevertheless, the bonds surrounding the manganese core are in general shorter if compared with the acac complexes. The angles deviate from the ideal bond angles for the octahedral complex – see Figure 4 below and its caption – which is most likely caused by the steric effect of the bulky mesityl substituents on the ligand. The O1−Mn1−O5 angle of 173.58(4)° in the z‐axis of the elongated bonds also leads to significant deviations of the angles between the apical oxygen atoms and those in the xy‐plane which are found in between 84.93(4)° for O3−Mn1−O1 and 96.44(4)° for O5−Mn1−O2. The dihedral angle of the four oxygen atoms O2−O4−O3−O6 in the xy‐plane around the manganese center deviates by 4.63° from planarity.

### Magnetic Susceptibility

**Figure 5:**
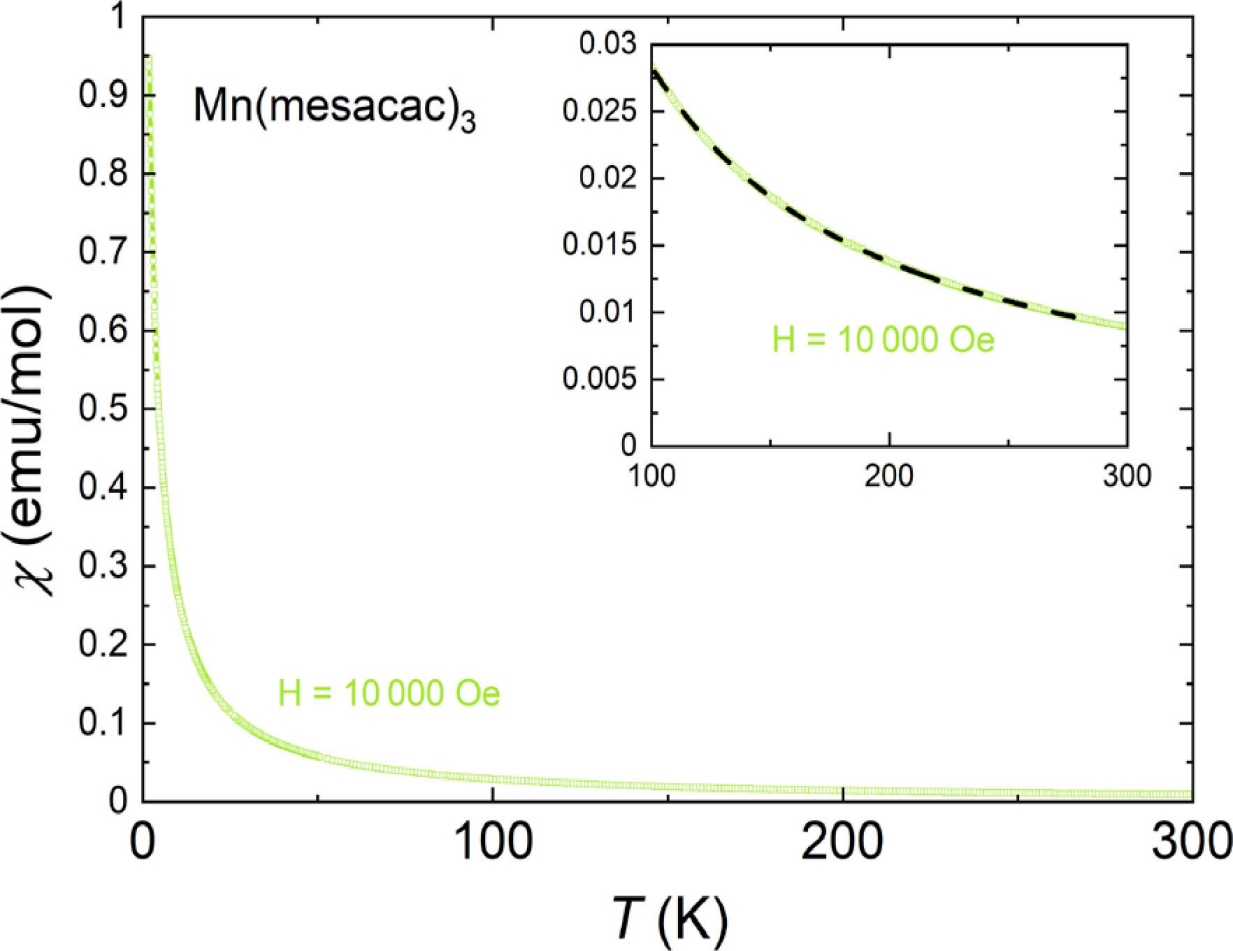
Molar magnetic susceptibility of pristine Mn(mesacac)_3_ *vs*. temperature at a field strength of 10000 Oe. The inset shows a Curie‐Weiss fit to the data.

Manganese (III) can in principle form complexes in three different spin states, depending on the ligands. One of the complexes which had been the subject of many investigations is the octahedral complex formed from the regular acetylacetonate.[^9a^, ^9c^, ^11a^, ^23^] The electronic structure of Mn(acac)_3_ has been recently reinvestigated in detail by a combination of photoemission spectroscopy, near‐edge X‐ray absorption fine structure (NEXAFS) spectroscopy and theoretical calculations.^[24]^ The study could confirm that the electronic configuration can be described as t^3^_2g_e^1^_g_ with S=2 (S corresponds to the total spin angular momentum quantum number). Compounds in a diamagnetic low‐spin state (t^4^_2g_e^0^_g_ with S=0) are rare, but in spin–crossover compounds the paramagnetic intermediate‐spin states (t^4^_2g_e^0^_g_ with S=1) have been found to be accessible.[^11^, ^12^] Mn(mesacac)_3_ displays paramagnetism of the manganese ions over the whole temperature range of our experiments. An effective moment of 4.85 $\mu_{B}$ was determined from a Curie‐Weiss fit $\chi =C/\left(T-\theta \right)+\chi_{0}$ together with a negligible Curie‐Weiss temperature (Figure 5). This agrees well with literature reference values of 4.90 to 5.00 $\mu_{B}$ for high‐spin Mn^3+^.^[25]^

**Figure 6:**
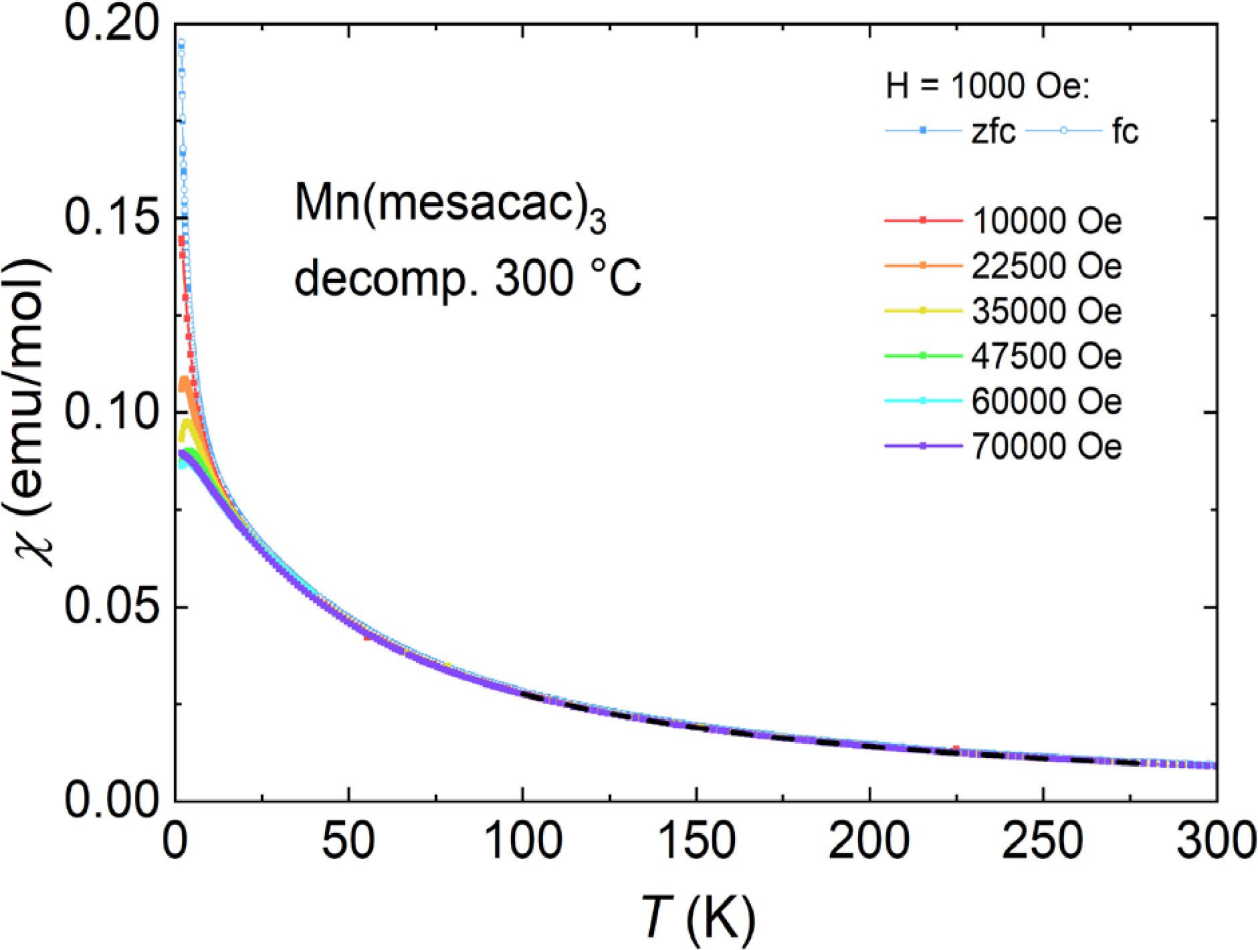
Molar magnetic susceptibility *vs*. temperature of the Mn(mesacac)_3_ sample after TGA measurements up to 300 °C. A Curie‐Weiss fit is plotted with a dashed line. Normalization is per mole of decomposed Mn(mesacac)_3_ or equivalently per mole of Mn.

As we had learned from the TGA measurement that at 270 °C a significant mass loss of 28 % could be observed which we attributed to a loss of the carbon fragment of the mesacac ligand, we heated another sample to 300 °C and repeated the magnetic measurement. Figure 6 shows that the magnetic properties of the partially decomposed Mn(mesacac)_3_ are Curie‐Weiss‐like – similar to those of the base material, pointing to a manganese ion magnetism.

A Curie‐Weiss fit to the data at high temperature gives an effective moment of (5.6±0.1) μ_B_ and a Curie‐Weiss temperature of −25 K. The obtained magnetic moment agrees with the literature range 5.65 to 6.10 $\mu_{B}$ for high‐spin Mn^2+^,^[25]^ while the negative sign of the Curie‐Weiss temperature indicates weak antiferromagnetic interactions between the Mn atoms. As can be seen at very low temperature, the susceptibility curves in a narrow range of magnetic fields (22500 and 35000 Oe) have an unexpected downwards curve while smaller and larger fields do not, pointing towards complex magnetic or electronic interactions in the decomposed Mn(mesacac)_3_.

**Figure 7:**
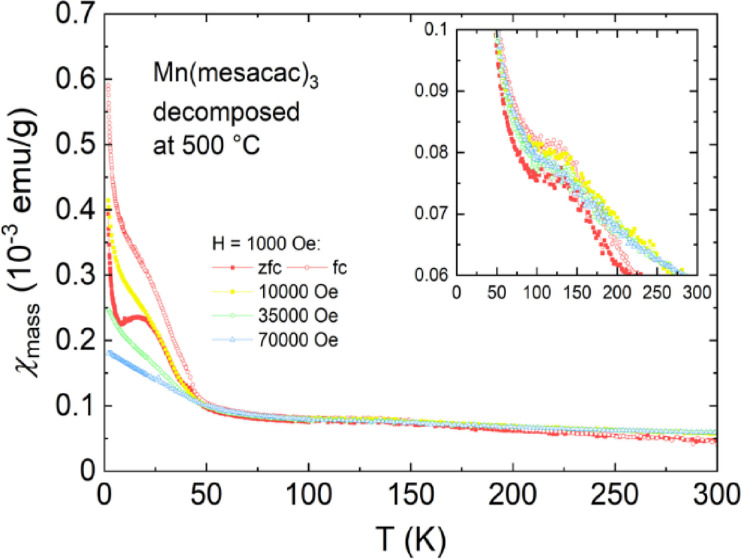
Mass magnetic susceptibility vs. temperature of the Mn(mesacac)_3_ sample after measuring a TGA up to 500 °C.

Another sample was heated to 500 °C to make sure that the complex is fully decomposed and to investigate the magnetic properties of the remaining material. The decomposition products of Mn(mesacac)_3_ display completely different magnetic properties compared to the other samples, which is consistent with our expectation (Figure 7). The magnetic susceptibility consists of two contributions, a magnetic‐field‐dependent contribution most prominent below 40 K and an almost magnetic‐field independent part dominant at higher temperatures. The upward curve below ca. 40 K and the corresponding magnetic‐field‐dependent behavior is due to the ferrimagnetism of Mn_3_O_4_ with a transition temperature of 42 K.[^26^, ^27^] The other manganese oxides are antiferromagnetic, with a *T_N_* =118 K for MnO,^[28]^ a *T_N_* =92 K for MnO_2_ ^[29]^ and a *T_N_* =80 K for Mn_2_O_3_.^[30]^

### Quantum Chemistry

To gain further insight into the electronic structure of the Mn(mesacac)_3_ complex we performed density functional theory (DFT) calculations on the different accessible spin states. For details on the calculations please see the computational details below and the Supporting Information. We tested different functionals on the double‐ and triple‐ξ level of theory and found that PBE0 and B3LYP gave reliable results, while the inclusion of dispersion effects predicts a contraction of the manganese‐oxygen bonds along the central xy‐plane, which is not in agreement with the data from the crystal structure. Both functionals significantly favor the high‐spin (HS) electron configuration by 7.9 kcal/mol (B3LYP) and 12.7 kcal/mol (PBE0) over the intermediate‐spin (IS) and by far over the low‐spin (LS) electron configuration (Table 1). The dispersion correction (D3BJ) enlarges the energy separation of the calculated B3LYP HS and IS states, but otherwise shows no real effect. Both functionals show a pronounced Jahn‐Teller effect in the high‐spin structures calculated with double‐ξ and triple‐ξ basis sets. We will discuss the results for the PBE0 functional in the paper, the corresponding data for the B3LYP functional is given in the supporting information (Tables S3 and S4).

**Table 1:** Free energies (in kcal/mol) of high‐spin (HS), intermediate‐spin (IS) and low‐spin state (LS) calculated by different DFT methods for Mn(mesacac)_3_.

	B3LYP dz	B3LYP dz D3BJ	PBE0 dz	PBE0 dz D3BJ
HS	0	0	0	0
IS	7.9	10.3	12.7	13.1
LS	44.2	43.8	52.2	51.5

The manganese oxygen bonds along the z‐axis of the molecule (axial bonds) were determined to be 2.116(1) Å and 2.145(1) Å in the crystal structure obviously due to solid–state effects while the DFT calculations found these bonds to both be 2.140 Å (dz) and 2.154 Å (tz) (Table 2). In general, all bond lengths calculated by the triple‐ξ basis sets are calculated to be longer than the corresponding double‐ξ basis sets, on average by 0.7 % (Table S4). The bonds in the xy‐plane (equatorial bonds) are also well reproduced in the high‐spin structure with the shorter (1.926 Å) and longer (1.940 Å) bonds on the same axis.

**Table 2:** Manganese‐oxygen bond lengths (given in Å) in the high‐spin (HS), intermediate‐spin (IS) and low‐spin state (LS) calculated by PBE0(tz) (PBE0(dz)) methods for Mn(mesacac)_3_.

	X‐Ray	HS	IS	LS
Mn−O1	2.116(1)	2.154 (2.140)	1.931 (1.920)	1.894 (1.886)
Mn−O2	1.9270(9)	1.926 (1.912)	1.924 (1.913)	1.951 (1.930)
Mn−O3	1.934(1)	1.940 (1.931)	1.914 (1.902)	1.911 (1.904)
Mn−O4	1.9209(9)	1.940 (1.931)	1.914 (1.902)	1.911 (1.904)
Mn−O5	2.145(1)	2.154 (2.140)	1.931 (1.920)	1.894 (1.886)
Mn−O6	1.9177(9)	1.926 (1.912)	1.924 (1.913)	1.952 (1.930)

**Figure 8:**
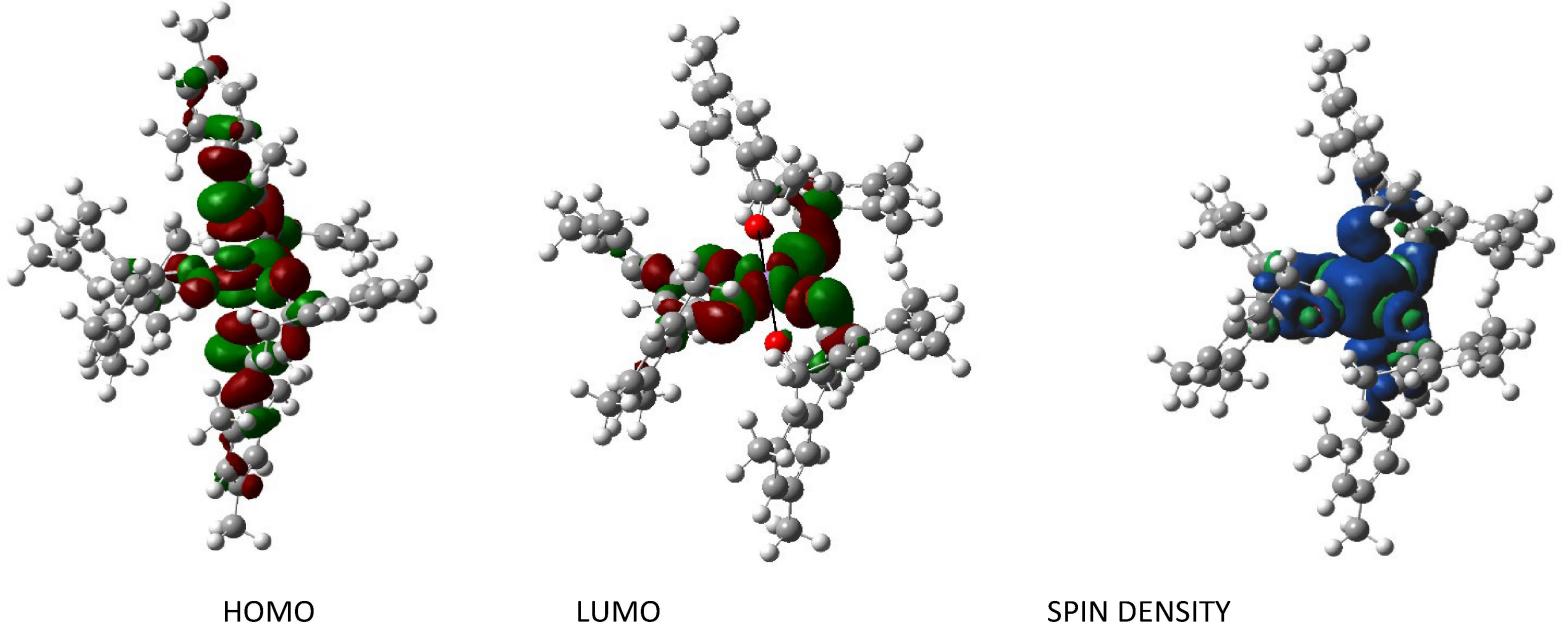
Calculated (PBE0/6‐31G(d)) frontier molecular orbitals (isoval=0.02) and spin density distribution (density=3.944) of the high‐spin Mn(mesacac)_3_.

The intermediate (1.910 Å–1.927 Å) and low‐spin (1.894 Å–1.952 Å) structures show significantly shorter bond lengths for the manganese‐oxygen bonds due to the different electron distribution which has also been visualized in spin density plots (Figures S3–S7) for the different spin states. An example is shown in Figure 8 together with the frontier molecular orbitals for the high‐spin state calculated by PBE0(dz). The spin density is clearly located on the central manganese atom and the surrounding oxygen atoms which can be considered as an octahedral coordination environment. All the electron density is roughly equally distributed over the manganese 3d‐orbitals interacting with the p‐orbitals of the oxygen atoms. As the O1−Mn−O5 angle along the z‐axis deviates 2.26° from the ideal 180 degrees this also has consequences for the O−Mn−O angles between the apical oxygen atoms (O1, O5) and the equatorial ones, which are in between 87.27° and 92.14°. The plane of the four equatorial oxygen atoms only shows a dihedral angle of 0.4° and the manganese center is right in the center of that plane. The O−Mn−O angles in the equatorial plane therefore show angles close to 90°.

The frontier molecular orbitals illustrate the Jahn‐Teller effect. The elongated z‐axis clearly dominates the highest occupied molecular orbital (HOMO). The electron density is distributed over the molecular orbitals (MO) along this axis with most of the electrons located on the manganese and oxygen core atoms.

Only weak interactions into the mesityl rings can be found, while the lowest unoccupied molecular orbital (LUMO) does not show any participation of the mesityl groups at all. Here, all the electron density is distributed over the MOs in the equatorial plane, built from the manganese atom and four oxygen atoms. The other two oxygen atoms in the z‐axis can clearly be seen and are not involved in the LUMO.

For comparison the corresponding orbitals calculated by B3LYP/6‐31G(d) are given in Figure S6, which agree very well with the findings described here. The findings from this DFT study also agree very well with the results of the magnetic susceptibility measurements, namely that the new complex Mn(mesacac)_3_ has a high‐spin ground state configuration. Independent of the choice of functional and basis set, the calculated energy differences between the different spin states are clearly favoring the high‐spin structure by at least 7.9 kcal/mol. Compared to the low‐spin state the energy differences are very large with at least 43.8 kcal/mol. The experimentally observed Jahn‐Teller elongation is represented in the high‐spin geometries of all functional and basis set combinations but is not found for any other spin state.

## Conclusions

In summary, we report the synthesis and characterization of a new octahedral manganese(III) complex with sterically very demanding 1,3‐bis(2,4,6‐trimethylphenyl)propane‐1,3‐dionato (mesacac) ligands. DFT calculations using the PBE0 functional together with basis sets of double‐ and triple‐ξ quality and the experimental results agree on a t^3^_2g_e^1^_g_ high‐spin state (S=2) of manganese showing a strong Jahn‐Teller elongation. The electronic state of manganese was confirmed by measurements of magnetic susceptibility, where a magnetic moment of 4.85 μ_B_ was experimentally determined for the metal center in the pristine molecule. The high thermal stability of the Mn^III^(mesacac)_3_ complex together with the interesting electronic structure provides great potential for a broad range of applications.

## Experimental Section

Solvents of at least 99.0 % purity were used in all reactions in this study. The 1,3‐bis(2,4,6‐trimethylphenyl)propane‐1,3‐dionato ligand was prepared according to a published procedure.^[31]^ All other chemicals were obtained from common suppliers and used without further purification. Elemental analyses were performed by the microanalytical laboratory of our institute on a Hekatech EA 3000 Euro Vector elemental analyzer. Melting points were determined by using a Wagner and Munz PolyTherm A system and are not corrected.

Absorption spectra were measured on a Perkin‐Elmer Lambda 365 UV/Vis spectrometer in DMF solutions with different analyte concentrations given with the spectra.

The thermogravimetric analyses were conducted with a Netzsch TG209 F1 Libra using Al_2_O_3_ crucibles under an argon atmosphere (sample chamber evacuated and refilled twice prior to analysis, 100 ml/min) and a heat rate of 5 K/min (25–500 °C).

**Synthesis of [Mn(mesacac)_3_]**. The tris(1,3‐bis(2,4,6‐trimethyl‐phenyl)propane‐1,3‐dionato‐κO^1^,κO^3^)) manganese(III) [Mn(mesacac)_3_] complex was prepared by adapting a published procedure^[18]^ from the manganese(II) acetate tetrahydrate (1.00 g, 4.08 mmol, 1 eq.) and the mesacacH ligand (3.78 g, 12.24 mmol, 3 eq.) in 150 ml ethanol in air (Scheme 1). The mixture was heated to 100 °C for two hours. During the reaction, the initially yellow color of the solution turned greenish‐brown. The dark product was filtrated, washed with diethyl ether and dried (3.13 g, 78 %). M.p. 279 °C. HRMS (ESI) m/z=977.4528 [M+H]^+^; 669.2752 [M‐mesacac]^+^. Anal. Calcd. for C_63_H_69_MnO_6_ (MW=977.17 g/mol): C, 77.44 %; H, 7.12 %. Found: C 77.09 %; H 7.20 %.

**Crystal Structure Determination**. Details of the X‐ray diffraction experiments on [Mn(mesacac)_3_], performed at 110 K, are given in the Supporting Information.

**Magnetism**. The magnetic properties were determined in a Quantum Design MPMS‐XL 7T SQUID magnetometer. The as‐synthesized samples were measured either as a coarse powder in a quartz tube or as a single small piece attached to a quartz paddle with GE Varnish. Due to the hygroscopic nature of the material, the more reliable method is to measure a single piece. To be able to use the material for additional tests, the samples previously measured in DTA experiments were scratched out of the DTA crucible with nonmagnetic tweezers and put into gelatine capsules, sealed shut with Kapton tape and measured in this form.

**Computational Methods**. The Gaussian 16, Rev. C.01^[32]^ program package was used to perform all quantum chemical calculations employing the hybrid functionals B3LYP^[33]^ and PBE0^[34]^ as established and reliable methods for the calculation of transition metal compounds^[35]^ together with the 6‐31G(d)^[36]^ and 6–311++G(d,p) basis sets.[^36h^, ^37^] Dispersion forces were simulated by using the D3 dispersion correction with Becke–Johnson damping (D3BJ).^[38]^ All given structures were optimized without any restrictions, employing the default grid (UltraFine). Frequency calculations were used to verify the nature of the stationary points as true minima, thermochemical data were taken from them at 298.15 K. If not stated otherwise, all discussed values are the ΔG_298_ values. For visualization GaussView^[39]^ and Molden^[40]^ have been used. Images were created with PyMOL^[41]^ and Mercury.^[42]^

## Conflict of interest

The authors declare no conflict of interest.

1

## Supporting information

As a service to our authors and readers, this journal provides supporting information supplied by the authors. Such materials are peer reviewed and may be re‐organized for online delivery, but are not copy‐edited or typeset. Technical support issues arising from supporting information (other than missing files) should be addressed to the authors.

Supporting Information

## Data Availability

The data that support the findings of this study are available in the supplementary material of this article.
