# Supplementary material for: Electronic Structure and Magnetic Properties of a High‐Spin MnIII Complex: [Mn(mesacac)3] (mesacac=1,3‐Bis(2,4,6‐trimethylphenyl)‐propane‐1,3‐dionato)
Source: Chemphyschem. 2022 Dec 14;24(2):e202200652. doi: 10.1002/cphc.202200652 (PMC10107892; doi:10.1002/cphc.202200652)
Supplement: Supplementary file 1 — Supporting Information [file CPHC-24-0-s001.pdf]

ChemPhysChem

Supporting Information

Electronic Structure and Magnetic Properties of a High-Spin Mn^{III} Complex: $[Mn(mesacac)_3]$ (mesacac = 1,3-Bis(2,4,6-trimethylphenyl)-propane-1,3-dionato)

Nina M. Strassner, Sergej Stipurin, Primož Koželj, Yuri Grin, and Thomas Strassner*

Table of contents:

General Procedures	S2
Electronic Absorption Spectroscopy (Figures S1, S2)	S2
Solid State Structure Determination (Tables S1, S2)	S3
DFT calculations (Tables S3, S4; Figures S3-S7)	S7
References	S14

General procedures

Solvents of at least 99.0 % purity were used in all reactions in this study. The precursor (mesacacH) for the 1,3-bis(2,4,6-trimethylphenyl)propane-1,3-dionato ligand (mesacac) was prepared according to a modified literature procedure.[1] All other chemicals were obtained from common suppliers and used without further purification.

ESI mass spectra were recorded on a Bruker Esquire LC mass spectrometer with an ion-trap detector, positive and negative ions were detected. HR-ESI mass spectra were recorded on a Waters Xevo G2-XS QTOF mass spectrometer.

Elemental analyses were performed at the TU Dresden by the microanalytical laboratory of our institute on a Hekatech EA 3000 Euro Vector elemental analyzer. Melting points were determined by using a Wagner and Munz PolyTherm A system and are not corrected.

Electronic Absorption Spectroscopy

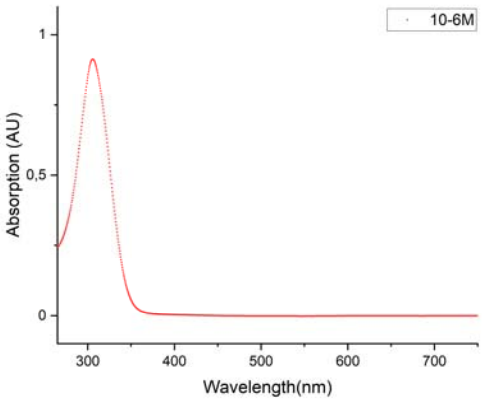

Figure S1. Absorption spectrum of $Mn(mesacac)_3$ (10^{-6} M in DMF) at room temperature.

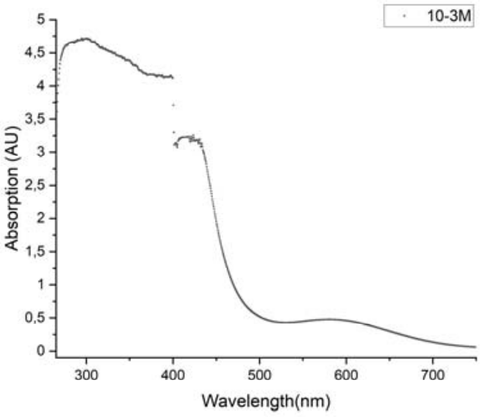

Figure S2. Absorption spectrum of $Mn(mesacac)_3$ (10^{-3} M in DMF) at room temperature.

Solid-structure determination

Preliminary examination and data collection for single crystals was carried out on a Bruker D8 VENTURE (KAPPA goniometer, PHOTON detector) single crystal-diffractometer equipped with an Oxford Cryosystem (Cryostream 800) cooling system at the window of a sealed x-ray tube using monochromated Mo-K_α radiation (λ = 0.71073 Å) (Incoatec IµS3.0 microfocus source equipped with multilayer optics). Intensity data were extracted using the APEX3 suite[2] including the SAINT software package.[3] The reflections were merged and corrected from Lorentz, polarization and decay effects and absorption correction was applied based on multiple scans.[4] The structure was solved by a combination of dual space[5], and direct methods with the aid of difference Fourier synthesis and were refined against all data using SHELXTL-XTMP.[6] Hydrogen atoms were assigned to ideal positions using the SHELXTL-XTMP riding model. All non-hydrogen atoms were refined with anisotropic displacement parameters. Full-matrix least-squares refinements were carried out by minimizing $\Sigma w(F_o^2-F_c^2)^2$ with the SHELXTL-XTMP weighting scheme. Neutral-atom scattering factors for all atoms and anomalous dispersion corrections for the non-hydrogen atoms were taken from the International Tables for Crystallography.[7] All calculations were performed with the APEX3 suite[2] including the SAINT software package[3], the SHELX program package[8] and PLATON.[9] For visualization ORTEP3[10] and Mercury [11] were used.

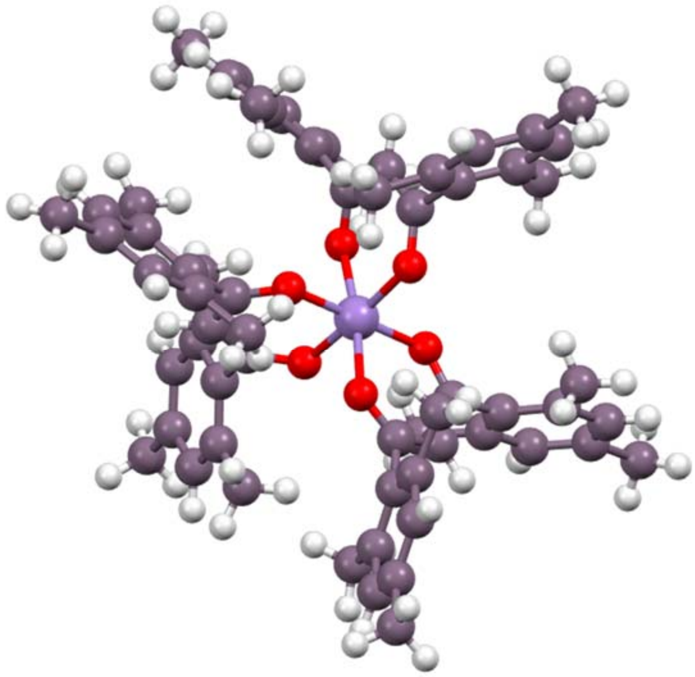

Table S1: Details of the solid state structure Mn(mesacac)$_3$

Identification code	2144201	
Empirical formula	$C_{63} H_{69} Mn O_6$	
Formula weight	977.12	
Temperature	110(2) K	
Wavelength	0.71073 Å	
Crystal system	Monoclinic	
Space group	$P2_1/n$	
Unit cell dimensions	a = 13.0479(9) Å	α= 90°.
	b = 15.6019(10) Å	β= 94.836(3)°.
	c = 26.7379(17) Å	γ = 90°.
Volume	5423.7(6) Å^3	
Z	4	
Density (calculated)	1.197 Mg/m^3	
Absorption coefficient	0.294 mm^{-1}	
F(000)	2080	
Crystal size	0.190 x 0.190 x 0.120 mm^3	
Theta range for data collection	2.010 to 28.336°.	
Index ranges	-17 <= h <= 17, -20 <= k <= 20, -35 <= l <= 35	
Reflections collected	639318	
Independent reflections	13494 [R(int) = 0.0543]	
Completeness to theta = 25.242°	99.9 %	
Refinement method	Full-matrix least-squares on F^2	
Data / restraints / parameters	13494 / 0 / 649	
Goodness-of-fit on F^2	1.022	
Final R indices [I>2sigma(I)]	R1 = 0.0347, wR2 = 0.0940	
R indices (all data)	R1 = 0.0409, wR2 = 0.1000	
Extinction coefficient	n/a	
Largest diff. peak and hole	0.340 and -0.458 e.Å^{-3}	

Table S2. Atomic coordinates (x 10^4) and equivalent isotropic displacement parameters (Å^2 x 10^3) for Mn(mesacac)$_3$. U(eq) is defined as one third of the trace of the orthogonalized U^{ij} tensor.

	x	y	z	U(eq)
Mn(1)	5605(1)	3928(1)	6756(1)	21(1)
O(1)	5135(1)	4723(1)	7335(1)	27(1)
C(1)	5460(1)	5473(1)	7421(1)	22(1)
O(2)	5977(1)	4946(1)	6405(1)	24(1)
C(2)	5883(1)	5993(1)	7057(1)	24(1)
O(3)	5133(1)	2996(1)	7154(1)	25(1)
C(3)	6081(1)	5715(1)	6582(1)	21(1)
O(4)	6951(1)	3914(1)	7107(1)	24(1)
C(4)	5373(1)	5778(1)	7948(1)	21(1)
C(5)	4402(1)	5756(1)	8141(1)	25(1)
O(5)	6148(1)	3027(1)	6233(1)	25(1)
C(6)	4344(1)	5905(1)	8651(1)	28(1)
O(6)	4283(1)	3937(1)	6383(1)	23(1)
C(7)	5216(1)	6054(1)	8975(1)	26(1)
C(8)	6164(1)	6092(1)	8775(1)	25(1)
C(9)	6258(1)	5969(1)	8263(1)	22(1)
C(10)	3433(1)	5580(1)	7807(1)	38(1)
C(11)	5129(1)	6172(1)	9531(1)	38(1)
C(29)	1548(1)	4074(1)	5227(1)	40(1)
C(28)	799(1)	3679(1)	5483(1)	46(1)
C(27)	1092(1)	3236(1)	5920(1)	44(1)
C(13)	6469(1)	6346(1)	6220(1)	22(1)
C(14)	5773(1)	6889(1)	5948(1)	25(1)
C(16)	7187(1)	7507(1)	5536(1)	31(1)
C(15)	6149(1)	7465(1)	5608(1)	30(1)
C(17)	7861(1)	6957(1)	5808(1)	30(1)
C(18)	7520(1)	6365(1)	6147(1)	26(1)
C(19)	4636(1)	6837(1)	6010(1)	33(1)
C(12)	7310(1)	6033(1)	8069(1)	29(1)
C(20)	7589(1)	8147(1)	5175(1)	42(1)
C(26)	2115(1)	3174(1)	6106(1)	33(1)
C(25)	2862(1)	3562(1)	5832(1)	24(1)
C(24)	5588(1)	2679(1)	5884(1)	21(1)
C(23)	4533(1)	2850(1)	5775(1)	26(1)

C(22)	3967(1)	3455(1)	6014(1)	21(1)
C(21)	8257(1)	5746(1)	6419(1)	36(1)
C(30)	2593(1)	4024(1)	5393(1)	29(1)
C(31)	2426(1)	2701(1)	6585(1)	48(1)
C(32)	-326(1)	3724(2)	5289(1)	75(1)
C(33)	3396(1)	4461(1)	5113(1)	37(1)
C(34)	6090(1)	2072(1)	5541(1)	21(1)
C(35)	5853(1)	1196(1)	5519(1)	23(1)
C(36)	6345(1)	678(1)	5187(1)	26(1)
C(37)	7046(1)	1008(1)	4872(1)	27(1)
C(38)	7254(1)	1882(1)	4897(1)	26(1)
C(39)	6790(1)	2421(1)	5226(1)	24(1)
C(40)	5107(1)	796(1)	5853(1)	31(1)
C(41)	7569(1)	433(1)	4517(1)	38(1)
C(42)	7001(1)	3371(1)	5230(1)	34(1)
C(43)	7244(1)	3460(1)	7493(1)	22(1)
C(44)	6601(1)	2914(1)	7740(1)	24(1)
C(45)	5582(1)	2736(1)	7571(1)	22(1)
C(46)	8359(1)	3525(1)	7665(1)	24(1)
C(47)	9089(1)	3358(1)	7318(1)	30(1)
C(48)	10128(1)	3361(1)	7491(1)	39(1)
C(49)	10459(1)	3544(1)	7985(1)	41(1)
C(50)	9725(1)	3745(1)	8314(1)	35(1)
C(51)	8676(1)	3741(1)	8165(1)	27(1)
C(52)	8800(1)	3175(1)	6771(1)	38(1)
C(53)	11595(1)	3539(2)	8161(1)	62(1)
C(54)	7921(1)	3990(1)	8536(1)	31(1)
C(55)	4929(1)	2193(1)	7881(1)	23(1)
C(56)	4605(1)	1381(1)	7709(1)	25(1)
C(57)	3978(1)	897(1)	8000(1)	28(1)
C(58)	3654(1)	1208(1)	8446(1)	31(1)
C(59)	3978(1)	2020(1)	8605(1)	32(1)
C(60)	4616(1)	2525(1)	8332(1)	28(1)
C(61)	4929(1)	3409(1)	8519(1)	40(1)
C(62)	4925(1)	1017(1)	7225(1)	35(1)
C(63)	2966(1)	672(1)	8748(1)	46(1)

DFT calculations

Computational details

The Gaussian 16, Rev. C.01[12] program package was used to perform all quantum chemical calculations employing the hybrid functionals B3LYP[13] and PBE0[14] as established and reliable methods for the calculation of transition metal compounds[15] together with the double-ξ(dz) 6-31G(d) [16] and triple-ξ(tz) 6-311++G(d,p) basis sets.[16h, 17] Additionally we also calculated tz single points (SP) on the dz geometries for comparison (6-311++G(d,p)//6-31G(d)). Dispersion forces were simulated by using the D3 dispersion correction with Becke-Johnson damping (D3BJ).[18] All given structures were optimized without any restrictions, employing the default grid (UltraFine). Frequency calculations on the double-ξ level of theory were used to verify the nature of the stationary points as true minima, thermochemical data were taken from them at 298.15 K. If not stated otherwise, all discussed values are the ΔG^{298} values. For visualization GaussView[19] and Molden[20] have been used. Images were created with PyMOL.[21] The coordinates of the optimized structures are given in a separate file.

Additionally we also conducted calculations with BP86[13a] and M06-2X[22], but both methods failed to provide reliable results for this system. According to BP86 the structure with intermediate spin is preferred over the high-spin structure by 10.1 kcal/mol (dz) or 8.6 kcal/mol (tz) while calculations with M06-2X did not even converge.

Calculations with dispersion correction led to a wrong description of the manganese-oxygen bond lengths in the xy-plane independent from the functional used (see Table S3).

XRay	B3LYP			PBE0		
	dz	dz D3BJ	tz	dz	dz D3BJ	tz
2.116(1)	2.157	2.145	2.174	2.140	2.147	2.154
1.9270(9)	1.927	1.904	1.944	1.912	1.898	1.926
1.934(1)	1.945	1.935	1.958	1.931	1.927	1.940
1.9209(9)	1.945	1.935	1.958	1.931	1.928	1.940
2.145(1)	2.157	2.145	2.174	2.140	2.145	2.154
1.9177(9)	1.927	1.904	1.944	1.912	1.899	1.926

Table S3: Comparison of bond lengths (in Å) for the high-spin state structure calculated with B3LYP and PBE0 functionals as well as double-ξ(dz), triple-ξ(tz) and dispersion correction (dz D3BJ).

B3LYP				PBE0			
dz (IS)	tz (IS)	dz (LS)	tz(LS)	dz (IS)	tz(IS)	dz (LS)	tz (LS)
1.931	1.941	1.954	1.983	1.902	1.910	1.930	1.911
1.928	1.941	1.901	1.983	1.902	1.910	1.886	1.911
1.922	1.941	1.901	1.912	1.913	1.927	1.886	1.952
1.922	1.941	1.954	1.928	1.920	1.921	1.930	1.951
1.931	1.941	1.917	1.928	1.913	1.927	1.904	1.894
1.928	1.941	1.917	1.912	1.920	1.921	1.904	1.894

Table S4: Comparison of bond lengths (in Å) for the intermediate (IS) and low spin (LS) state calculated with B3LYP and PBE0 functionals as well as double-ξ(dz) and triple-ξ(tz) basis sets.

DFT calculated frontier molecular orbitals and spin density distributions

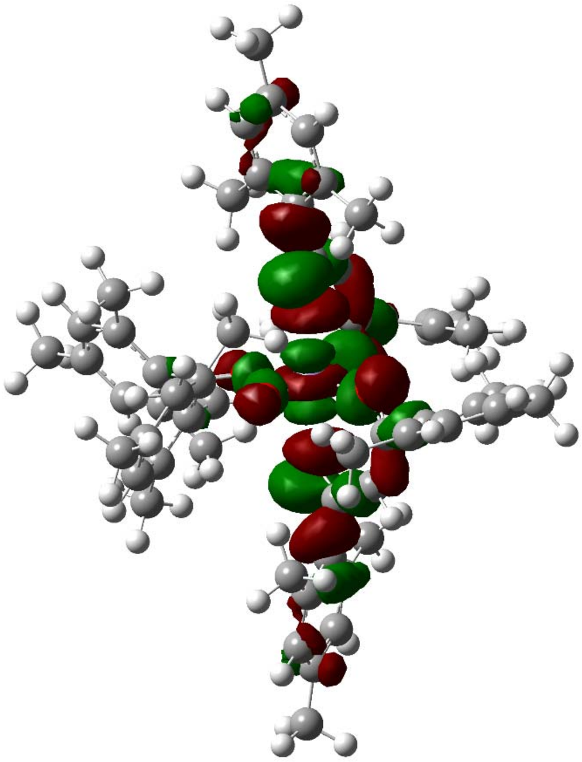

HOMO

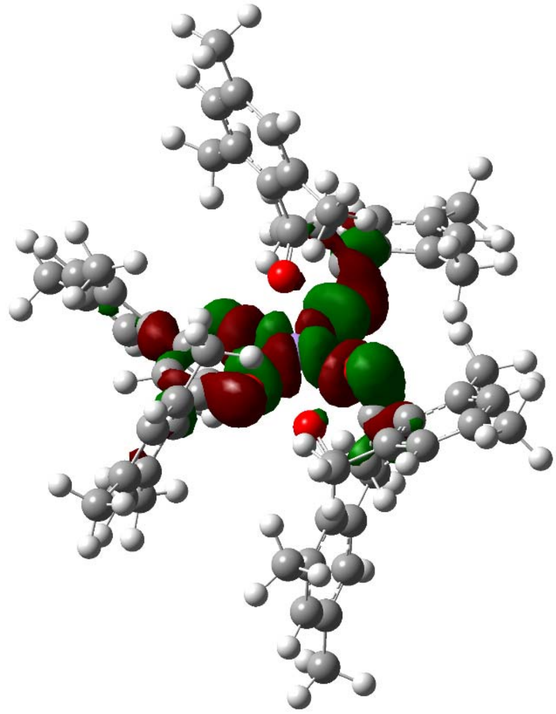

LUMO

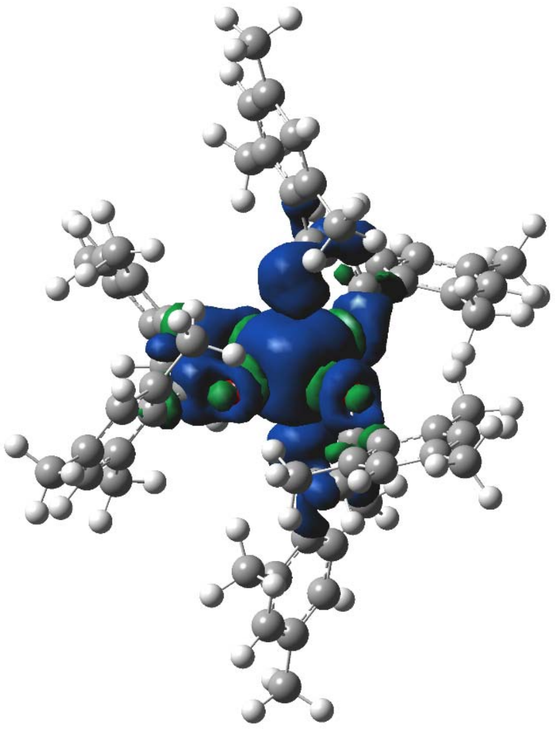

SPIN DENSITY

Figure S3. Calculated (PBE0/6-31G(d)) frontier molecular orbitals (isoval = 0.02) and spin density distribution (density = 3.944) of the high spin Mn(mesacac)$_3$.

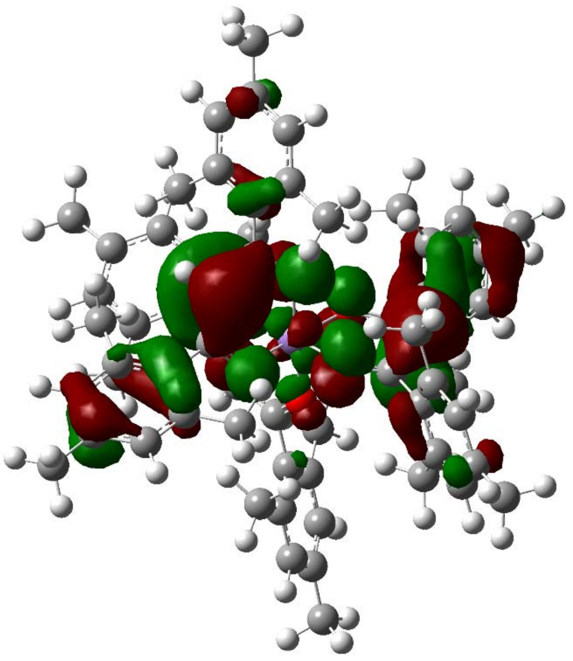

HOMO

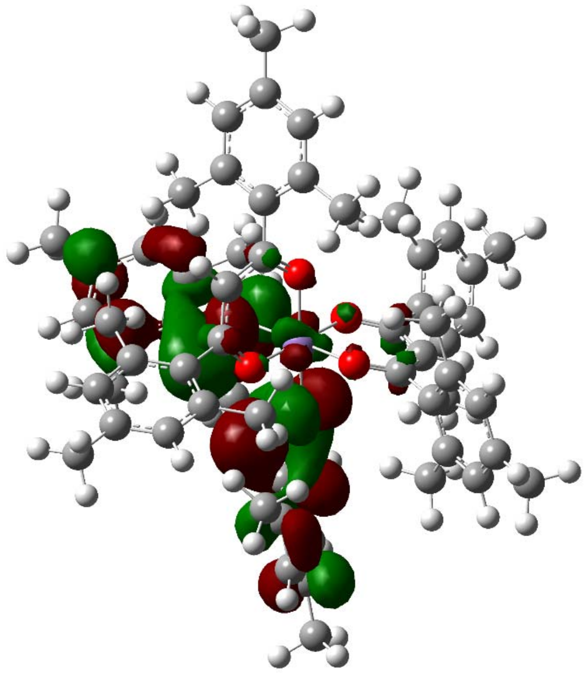

LUMO

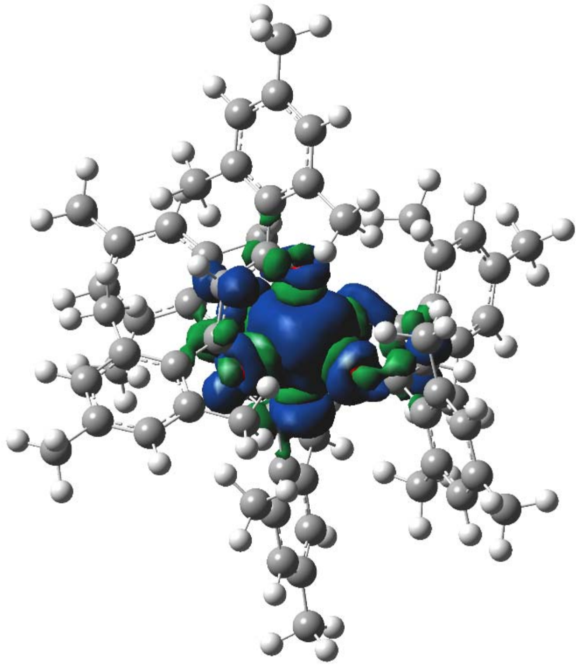

SPIN DENSITY

Figure S4. Calculated (PBE0/6-31G(d)) frontier molecular orbitals (isoval = 0.02) and spin density distribution (density = 2.068) of the intermediate spin Mn(mesacac)$_3$.

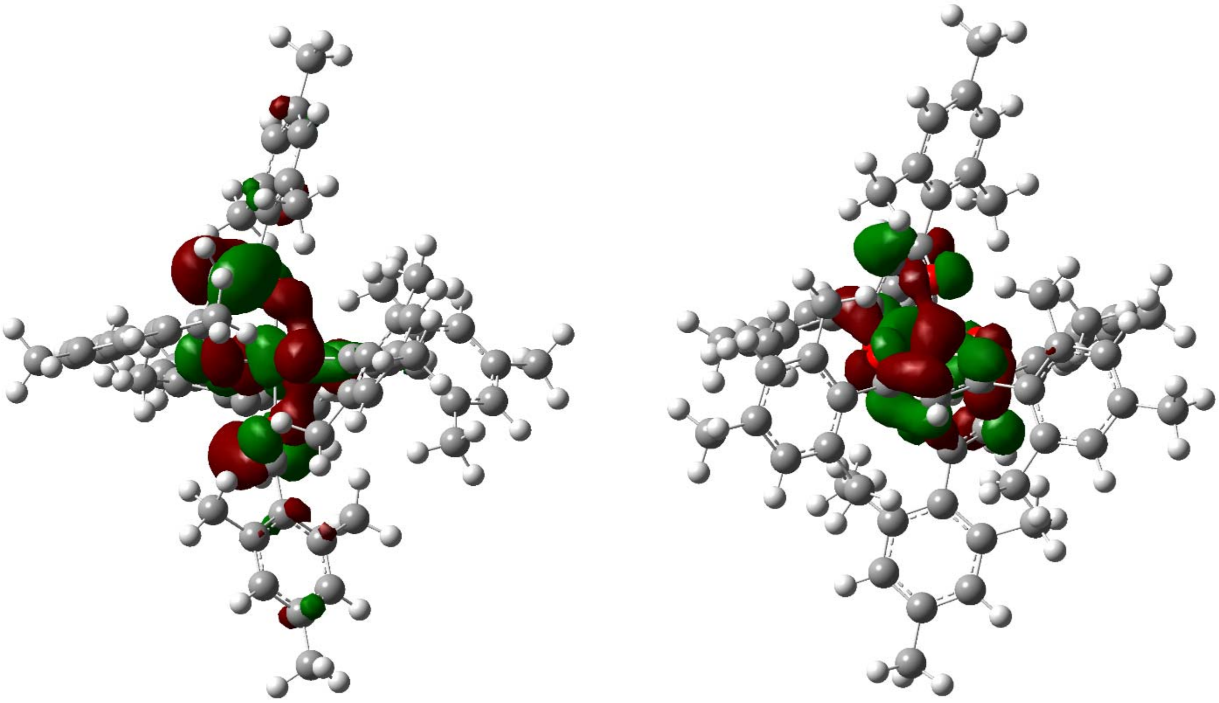

HOMO **LUMO**

Figure S5. Calculated (PBE0/6-31G(d)) frontier molecular orbitals (isoval = 0.02) of the low spin Mn(mesacac)$_3$.

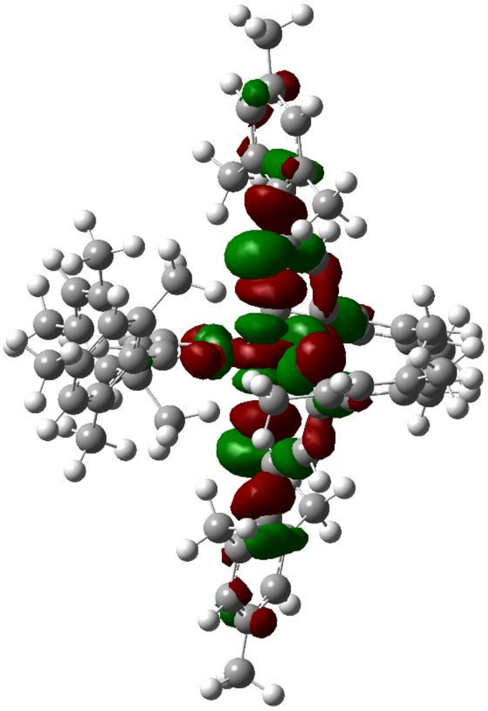

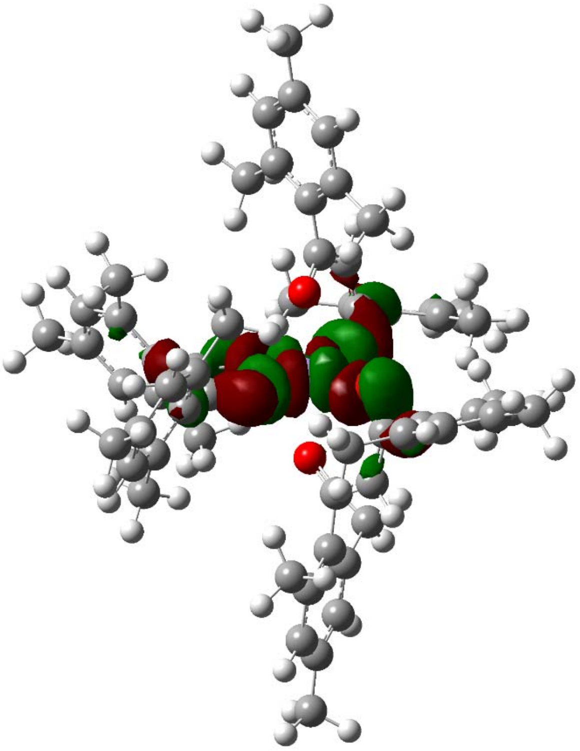

HOMO **LUMO**

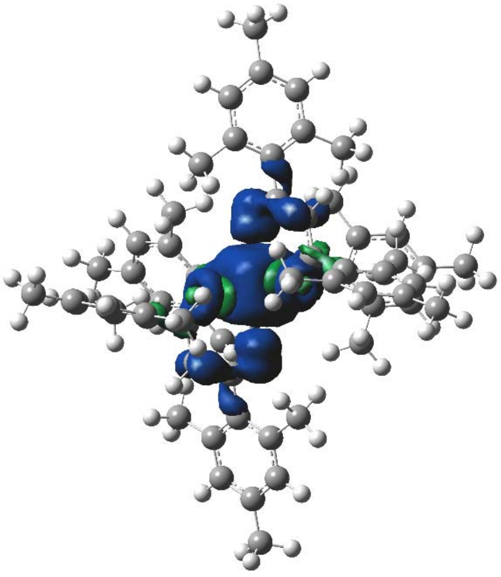

SPIN DENSITY

Figure S6. Calculated (B3LYP/6-31G(d)) frontier molecular orbitals (isoval = 0.02) and spin density distribution (density = 3.792) of the high spin Mn(mesacac)$_3$.

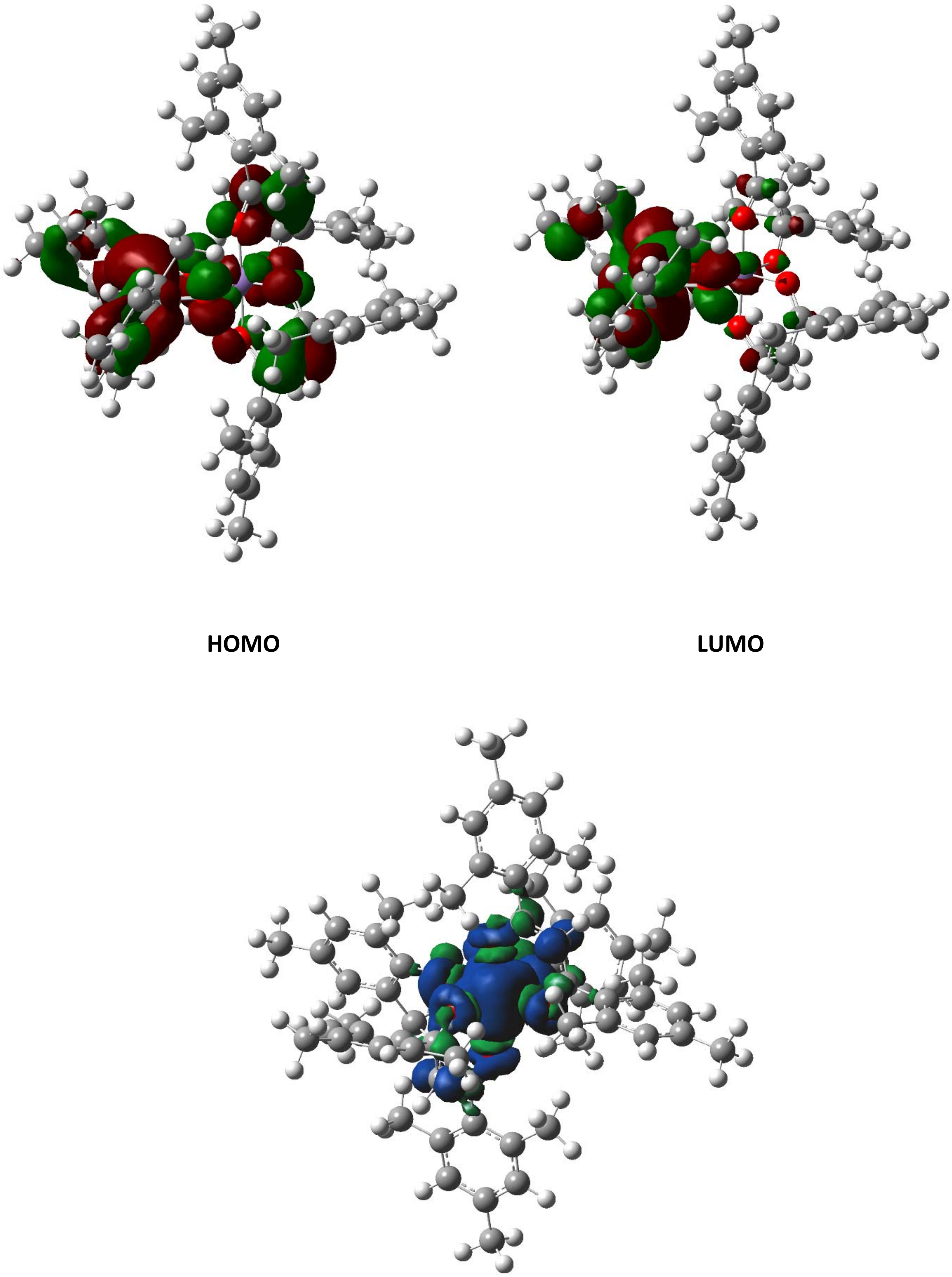

HOMO **LUMO**

SPIN DENSITY

Figure S7. Calculated (B3LYP/6-31G(d)) frontier molecular orbitals (isoval = 0.02) and spin density distribution (density = 2.036) of the intermediate spin Mn(mesacac)$_3$.

References

[1] C. Zhang, P. Yang, Y. Yang, X. Huang, X.-J. Yang, B. Wu, *Synth. Commun.* **2008**, *38*, 2349-2356.

[2] *Bruker Crystallographic Suite APEX3, v2017.3*, Bruker AXS Inc., Madison, Wisconsin, USA, **2017**.

[3] *Bruker Integration Engine SAINT V8.38A*, Bruker AXS Inc., Madison, Wisconsin, USA, **2017**.

[4] G. M. Sheldrick, *SADABS Multi Scan Absorption, SADABS-2016/2*, University of Goettingen, Goettingen, Germany, **2016**.

[5] G. M. Sheldrick, *Acta Cryst. C* **2015**, *71*, 3-8.

[6] G. M. Sheldrick, *Acta Cryst. A* **2015**, *71*, 3-8.

[7] Iucr, A. J. C. Wilson, *International Tables for Crystallography, Volume C: Mathematical, physical and chemical tables*, Kluwer Academic Publishers, Dordrecht, Boston, London, **1992**.

[8] G. M. Sheldrick, *Acta cryst.A* **2008**, *64*, 112-122.

[9] A. L. Spek, *Acta cryst. D* **2009**, *65*, 148-155.

[10] a) L. J. Farrugia, *J. Appl. Cryst.* **1997**, *30*, 565-565; b) M. N. Burnett, C. K. Johnson, *ORTEP-3 for Windows v2014.1*, Oak Ridge National Laboratory, Oak Ridge, TN, USA, **2014**.

[11] C. F. Macrae, I. Sovago, S. J. Cottrell, P. T. A. Galek, P. McCabe, E. Pidcock, M. Platings, G. P. Shields, J. S. Stevens, M. Towler, P. A. Wood, *J. Appl. Crystallogr.* **2020**, *53*, 226-235.

[12] M. J. Frisch, G. W. Trucks, H. B. Schlegel, G. E. Scuseria, M. A. Robb, J. R. Cheeseman, G. Scalmani, V. Barone, G. A. Petersson, H. Nakatsuji, X. Li, M. Caricato, A. V. Marenich, J. Bloino, B. G. Janesko, R. Gomperts, B. Mennucci, H. P. Hratchian, J. V. Ortiz, A. F. Izmaylov, J. L. Sonnenberg, Williams, F. Ding, F. Lipparini, F. Egidi, J. Goings, B. Peng, A. Petrone, T. Henderson, D. Ranasinghe, V. G. Zakrzewski, J. Gao, N. Rega, G. Zheng, W. Liang, M. Hada, M. Ehara, K. Toyota, R. Fukuda, J. Hasegawa, M. Ishida, T. Nakajima, Y. Honda, O. Kitao, H. Nakai, T. Vreven, K. Throssell, J. A. Montgomery Jr., J. E. Peralta, F. Ogliaro, M. J. Bearpark, J. J. Heyd, E. N. Brothers, K. N. Kudin, V. N. Staroverov, T. A. Keith, R. Kobayashi, J. Normand, K. Raghavachari, A. P. Rendell, J. C. Burant, S. S. Iyengar, J. Tomasi, M. Cossi, J. M. Millam, M. Klene, C. Adamo, R. Cammi, J. W. Ochterski, R. L. Martin, K. Morokuma, O. Farkas, J. B. Foresman, D. J. Fox, *Gaussian16 Rev. C.01.* Wallingford, CT, **2016**.

[13] a) A. D. Becke, *Phys. Rev. A* **1988**, *38*, 3098-3100; b) A. D. Becke, *J. Chem. Phys.* **1993**, *98*, 1372-1377; c) B. Miehlich, A. Savin, H. Stoll, H. Preuss, *Chem. Phys. Lett.* **1989**, *157*, 200-206; d) P. J. Stephens, F. J. Devlin, C. F. Chabalowski, M. J. Frisch, *J. Phys. Chem.* **1994**, *98*, 11623-11627; e) S. H. Vosko, L. Wilk, M. Nusair, *Can. J. Phys.* **1980**, *58*, 1200-1211.

[14] a) C. Adamo, V. Barone, *J. Chem. Phys.* **1999**, *110*, 6158-6170; b) J. P. Perdew, K. Burke, M. Ernzerhof, *Phys. Rev. Lett.* **1996**, *77*, 3865-3868; c) J. P. Perdew, K. Burke, M. Ernzerhof, *Phys. Rev. Lett.* **1997**, *78*, 1396-1396.

[15] Y. Zhao, D. G. Truhlar, *Acc. Chem. Res.* **2008**, *41*, 157-167.

[16] a) J.-P. Blaudeau, M. P. McGrath, L. A. Curtiss, L. Radom, *J. Chem. Phys.* **1997**, *107*, 5016-5021; b) R. Ditchfield, W. J. Hehre, J. A. Pople, *J. Chem. Phys.* **1971**, *54*, 724-728; c) M. M. Francl, W. J. Pietro, W. J. Hehre, J. S. Binkley, M. S. Gordon, D. J. DeFrees, J. A. Pople, *J. Chem. Phys.* **1982**, *77*, 3654-3665; d) M. S. Gordon, *Chem. Phys. Lett.* **1980**, *76*, 163-168; e) P. C. Hariharan, J. A. Pople, *Theor. chim. acta* **1973**, *28*, 213-222; f) G. A. Petersson, A. Bennett, T. G. Tensfeldt, M. A. Al-Laham, W. A. Shirley, J. Mantzaris, *J. Chem. Phys.* **1988**, *89*, 2193-2218; g) V. A. Rassolov, J. A. Pople, M. A. Ratner, T. L. Windus, *J. Chem. Phys.* **1998**, *109*, 1223-1229; h) M. J. Frisch, J. A. Pople, J. S. Binkley, *J. Chem. Phys.* **1984**, *80*, 3265-3269.

[17] a) T. Clark, J. Chandrasekhar, G. W. Spitznagel, P. V. R. Schleyer, *J. Comput. Chem.* **1983**, *4*, 294-301; b) R. Krishnan, J. S. Binkley, R. Seeger, J. A. Pople, *J. Chem. Phys.* **1980**, *72*, 650-654; c) A. D. McLean, G. S. Chandler, *J. Chem. Phys.* **1980**, *72*, 5639-5648; d) L. A. Curtiss, M. P. McGrath, J.-P. Blaudeau, N. E. Davis, R. C. Binning, L. Radom, *J. Chem. Phys.* **1995**, *103*, 6104-6113.

[18] S. Grimme, S. Ehrlich, L. Goerigk, *J. Comput. Chem.* **2011**, *32*, 1456-1465.

[19] R. K. Dennington, T.; Millam, J., Gaussview, Vers. 5.0.8. *Semichem Inc., Shawnee Mission KS* **2009**.

[20] G. Schaftenaar, J. H. Noordik, *J. Comp.-Aid. Mol. Design* **2000**, *14*, 123-134.

[21] Schrodinger, LLC, The PyMOL Molecular Graphics System, Version 2.4.1. **2015**.

[22] Y. Zhao, D. G. Truhlar, *Theor. Chem. Acc.* **2008**, *120*, 215-241.
